# Multiple Myeloma in 2023 Ways: From Trials to Real Life

**DOI:** 10.3390/curroncol30110705

**Published:** 2023-11-03

**Authors:** Manlio Fazio, Vittorio Del Fabro, Nunziatina Laura Parrinello, Alessandro Allegra, Uroš Markovic, Cirino Botta, Fabrizio Accardi, Iolanda Donatella Vincelli, Salvatore Leotta, Federica Elia, Benedetta Esposito, Bruno Garibaldi, Gabriele Sapuppo, Alessandra Orofino, Alessandra Romano, Giuseppe A. Palumbo, Francesco Di Raimondo, Concetta Conticello

**Affiliations:** 1 Post-Graduation School of Haematology, University of Catania, A.O.U. ‘Policlinico-San Marco’, 95123 Catania, Italy; manliofazio@hotmail.it (M.F.); benedetta.esposito89@gmail.com (B.E.); brunga93@gmail.com (B.G.); g.sap.95@gmail.com (G.S.); aleoro.91@gmail.com (A.O.); diraimon@unict.it (F.D.R.); 2 Division of Haematology and BMT, A.O.U. ‘Policlinico-San Marco’, 95123 Catania, Italy; vdelfabro@yahoo.it (V.D.F.); parrinellolaura@gmail.com (N.L.P.); urosmarkovic09041989@gmail.com (U.M.); leotta3@yahoo.it (S.L.); fede.elia31@gmail.com (F.E.); 3 Division of Haematology, Department of Human Pathology in Adulthood and Childhood “Gaetano Barresi”, University of Messina, 98125 Messina, Italy; aallegra@unime.it; 4 Department of Health Promotion, Mother and Child Care, Internal Medicine and Medical Specialties “G. D’Alessandro”, University of Palermo, 90127 Palermo, Italy; cirino.botta@unipa.it; 5 Department of Hematology I, Azienda Ospedaliera Ospedali Riuniti Villa Sofia-Cervello, 90146 Palermo, Italy; accardi.fabrizio@gmail.com; 6 Haematology Unit, Haemato-Oncology and Radiotherapy Department, Grande Ospedale Metropolitano “Bianchi-Melacrino-Morelli”, 89124 Reggio Calabria, Italy; donatella.vincelli@gmail.com; 7 Dipartimento di Specialità Medico-Chirurgiche, CHIRMED, Sezione di Ematologia, Università degli Studi di Catania, 95131 Catania, Italy; sandrina.romano@gmail.com; 8 Dipartimento di Scienze Mediche, Chirurgiche e Tecnologie Avanzate “G.F.Ingrassia”, University of Catania, 95131 Catania, Italy; palumbo.ga@gmail.com

**Keywords:** multiple myeloma, minimal residual disease, standard of care, daratumumab, autologous stem cell transplantation, frailty, relapsed-refractory disease, sequencing, bispecific antigens, CAR-T cells therapy, real-life

## Abstract

Multiple myeloma is a chronic hematologic malignancy that obstinately tends to relapse. Basic research has made giant strides in better characterizing the molecular mechanisms of the disease. The results have led to the manufacturing of new, revolutionary drugs which have been widely tested in clinical trials. These drugs have been approved and are now part of the therapeutic armamentarium. As a consequence, it is essential to combine what we know from clinical trials with real-world data in order to improve therapeutic strategies. Starting with this premise, our review aims to describe the currently employed regimens in multiple myeloma and compare clinical trials with real-life experiences. We also intend to put a spotlight on promising therapies such as T-cell engagers and chimeric antigen receptor T-cells (CAR-T) which are proving to be effective in changing the course of advanced-stage disease.

## 1. Introduction

Multiple Myeloma (MM) is the second most common hematologic malignancy worldwide, with an estimated annual incidence of 7.1 per 100,000 men and women. The main feature of the disease is B-cells and plasma-cells (PCs) proliferation in the bone marrow (BM) and in extramedullary (EM) organs, with the secretion of monoclonal immunoglobulins described as monoclonal (M) protein. In 10–20% of cases, patients are asymptomatic and present with ≥10% PCs in the BM, a condition described as smoldering myeloma (SM) which does not require any treatment. On the other hand, MM is described as an active disease when a patient develops several pathological conditions and organ damage such as hypercalcemia, lytic bone lesions, anemia, renal insufficiency, and hyper-viscosity. This severe clinical picture needs immediate treatment and supportive care. Treatment options have evolved throughout the years, incorporating novel drugs in well-established therapeutic backbones. Despite these significant advancements, the selection of mutated clones leads to subsequent relapses. Employing a combination of 3 or 4 agents in early treatments leads to multi-drug resistance at an earlier stage. Patients who relapse after 2 immunomodulatory drugs, 2 proteosome inhibitors, and an anti-CD38 antibody are defined as penta-refractory and triple-class refractory and are characterized by a very dismal prognosis [1]. Various strategies can be employed in this very challenging category of patients with no particular standard of care. Particular mention should be made to T-cell therapy which has obtained surprising results in terms of survival and achievement of minimal residual disease. The purposes of this review are to summarize existing literature on current treatment strategies at diagnosis and relapse according to transplant eligibility or not, emphasize the importance of real-world experiences to consolidate data from clinical trials and find new food for thought, keep an eye on future therapeutic scenarios which will progressively change the approach to the disease, and reaffirm the role of minimal residual disease (MRD) in predicting outcome and directing therapy and remarking the necessity to create therapeutic guidelines for major unmet clinical needs such as extramedullary disease.

## 2. Methods

Publications of interest were searched on the PubMed database from January 2000 to October 2023. The following search terms were employed in the research: [minimal residual disease in multiple myeloma AND (next generation flow OR positron emission tomography)], [newly diagnosed multiple myeloma AND (standard of care AND transplant eligible OR transplant ineligible)], [relapsed refractory myeloma AND lenalidomide OR (clinical trials OR real-life/world)], [multiple myeloma AND (bispecific antibodies OR CAR-T therapy)], [multiple myeloma AND (anti-BCMA AND/OR anti-GPRC5D targeting)], [multiple myeloma AND (extramedullary disease OR plasmacytoma)], [extramedullary multiple myeloma AND (clinical trials OR real-life)]. Supplemental material was extrapolated from abstracts reported in prominent conferences that took place in 2022 and 2023: American Society of Hematology (ASH-New Orleans 2022), American Society of Clinical Oncology (ASCO-Chicago 2023), European Myeloma Network (EMN-Amsterdam 2023). Full-text articles written in the English language and considered relevant for inclusion were carefully reviewed with particular attention to progression-free/overall survival (PFS/OS) clinical and laboratory response, and (MRD).

## 3. Discussion

### 3.1. Minimal Residual Disease 

#### 3.1.1. Focus on Plasma Cells

Minimal Residual Disease (MRD) identifies the persistence of a minimal number of malignant cells in the bone marrow (BM) when a patient is in remission after a treatment. The evaluation is based on the detection of plasma cells (PCs) in the BM [2] through flow cytometry or next-generation sequencing (NGS) and should be integrated with imaging tests like positron-emission tomography-computed tomography (PET-CT) [2]

MRD-negative patients present a higher progression-free survival (PFS) and overall survival (OS). In addition, MRD could allow us to understand in advance if a therapy is effective or should potentially be changed. For this reason, it is emerging the concept that MRD should be incorporated in clinical trials as a surrogate endpoint of outcome (PFS and OS) or should itself be considered as a new primary goal of MM treatment [3]. 

To emphasize the importance of MRD, in 2016 the International Myeloma Working Group (IMWG) introduced new response subcategories: sustained MRD-negativity, Flow MRD negativity, Sequencing MRD negativity, and Imaging positive MRD-negativity. The term Flow-MRD negativity refers to “*the absence of phenotypically aberrant plasma cells documented by Next Generation Flow (NGF) on bone marrow aspirates using the Euro Flow standard operation procedure for MRD detection in multiple myeloma (or validated equivalent method) with a minimum sensitivity of 1 in 10^5^ nucleated cells or higher*”. 

Compared to NGS, NGF presents with a similar sensitivity (1 × 10^6^) but a more rapid execution (3–4 h) and does not require the collection of a starting sample in order to identify the dominant clone [4].

The NGF-MRD method has a limit of detection (LOD) and quantification (LOQ) of respectively 20 and 50 neoplastic cells/number of viable nucleated cells × 100. On these bases, a minimum of 2 × 10^6^ events should be acquired to reach the desired sensitivity of 10^−5^.

The antibodies usually employed to discriminate normal from abnormal PCs are directed against the following markers: CD19, CD56, CD20, CD28, CD27, CD81, CD117, CD200, cytoplasmic (Cy) immunoglobulin (Ig) κAPC/CyIgλAPC-H7. OneFlow panels do not include CD200, while EuroFlow panels do not include CD20, CD28 and CD200. Normal PCs are CD38+, 138+,81+,27+,56−, 117− and both Cy IgK and Igλ, while abnormal PCs are CD38+, CD 81 and 27 weak/negative, 56+, 117+ and Cy IgK or Igλ. The panel has been recently extended, by some groups, with the introduction of antibodies directed against target antigens like CD269 [B-cell maturation antigen (BCMA)], CD319 [SLAM family member-7 (SLAMF-7)], and CD279 [Programmed cell death protein 1 (PD-1)]. Lastly, markers available for alternative gating are CD38ME, IRF4, P63(VS38c), CD48, CD54, CD86, CD150, CD229, and CD272, for patients treated with daratumumab and isatuximab [5].

At present, the research of neoplastic PCs in peripheral blood is emerging as a noninvasive strategy for monitoring disease dynamics [6] and we need to clarify if the quantity of circulating tumor plasma cells (CTPCs) is directly proportional to the neoplastic PCs in the BM or if they represent a distinct population with a different biologic significance [7]. In this respect, the presence of CTPCs in patients with MGUS (which present a lower monoclonal component), seems to suggest that the % of these cells in PB shouldn’t be completely ascribed to the entity of BM disease but also to their ability to elude control mechanisms. For example, it has been noticed that the expression of the adhesion molecule C-X-C chemokine receptor type 4 (CXCR4) is higher in circulating PCs than in those that remain in the BM [8]. Lastly, we are trying to understand if the presence of CTPCs can also be a reflection of extramedullary disease (EMM) [7]. 

#### 3.1.2. Focus on the Role of ^18^f-Fdg Pet/Ct in Defining Mrd

Bone marrow MRD assessment can easily lead to false-negative results due to the uneven PC infiltration pattern. Moreover, MRD negativity in the BM does not necessarily match with MRD negativity in the extramedullary compartment. Recent studies have also demonstrated the coexistence of different clones in BM and extramedullary sites which proves the spatial heterogenicity of the disease [9]. 

Functional body imaging techniques, such as [18]-2-fluoro-2-deoxy-D-glucose PET/CT and magnetic resonance imaging (MRI) help to define the tumor burden beyond osteolytic lesions and identify focal lesions (FLs) which suggest para-medullary and extramedullary disease (ED). Compared with morphologic classic T1 and T2 weighted MRI sequences, PET/CT allows to detect of metabolically active MM lesions with fewer false positive images [10].

PET/CT image interpretation typically relies on the standardized uptake volume (SUV) maximum (max) and ^18^F-FDG is the most commonly used tracer. In fact, tumor cells increase glucose uptake and lactate production (Warburg Effect) [11]. Reduction in focal lesions (FLs) metabolism has shown to have a prognostic meaning and a complete normalization of PET/CT findings after induction therapy is associated with higher PFS and OS [12]. In a retrospective study of both ASCT-eligible and ineligible patients, PET/CT was evaluated at diagnosis and after treatment. Nearly 29% of CRs were still PET/CT positive. At 5 years of FU, these patients had a shorter OS compared to those with no more detectable lesions (70% vs. 90%) [13]. 

Moreover, in a study published by Rasche et al. in 2019, 12% of MRD-negative patients, defined by flow cytometry, still had PET/CT positivity. This condition correlated with shorter PFS [14]. Furthermore, double negativity (both in the BM and at PET/CT) can be considerate a surrogate for outcome prediction, while double positivity or discordant results between the 2 methods should be considered for categorizing patients in specific prognostic classes [12]. 

Beyond ^18^F-FDG, new PET/CT tracers such us CXCR4 or CD38 have been preliminarily investigated and might prove to be valid molecular imaging biomarkers [15].

Establishing internationally standardized interpretation criteria for PET/CT images has been a primary issue for years. In 2021, the Deauville score (DS) criteria were definitively applied to MM PET/CT evaluation. Complete metabolic response has been defined as an *^18^F-FDG uptake minus the liver activity in BM sites and FLs previously involved (including extramedullary and para-medullary disease (DS score 1–3)* [16]. 

In 2022 PET/CT has been listed as the standard image technique to evaluate response in MM by the IMWG [17].

In conclusion, it is fundamental to combine cell and molecular-based MRD assessments with ^18^F-FDG PET/CT evaluation in order to stratify MRD at different levels and better predict patients’ response and outcome.

### 3.2. Excursus on Treatment: At Diagnosis

#### 3.2.1. Transplant-Eligible Patients

The standard of care (SOC) in Italy for first-line therapy in young transplant eligible (TE) patients was previously represented by the triplet: immunomodulatory imide drugs (IMiDs)-proteosome inhibitors (PIs)-corticosteroids [18], and has now been converted into a quadruplet with the addition of monoclonal antibody (mAb) daratumumab. 

**Daratumumab** (DARA) is an IgG1κ human monoclonal antibody (MAb) that binds to the CD38 protein, a surface protein that is expressed on MM cells, and inhibits the in vivo growth of CD38-expressing tumor cells. DARA acts through different mechanisms: antibody-dependent cellular cytotoxicity (ADCC) or phagocytosis (ADCP), complement-dependent cytotoxicity (CDC), direct apoptosis, and immunomodulation on microenvironment. Its action is enhanced by other compounds, such as immunomodulatory agents (IMiDs) and proteasome inhibitors (PI) [19].

The principle aim of induction therapy is to reach the deepest response which will allow the patient to autologous stem cell transplant (ASCT) and delay the relapse. According to this, the 2021 EHA-ESMO clinical practice guidelines recommend bortezomib-lenalidomide-dexamethasone (VRd) or daratumumab-bortezomib-thalidomide-dexamethasone (D-VTd) as first therapeutic options in TE patients and, if not possible, VTd or bortezomib-cyclophosphamide-dexamethasone (VCd) followed by ASCT with eventual maintenance therapy [20].

The multicenter, phase III randomized clinical trial (RCT) MMY-3006 **CASSIOPEIA** (PART 1 and 2) was pivotal in demonstrating the superiority of D-VTd quadruplet in newly diagnosed (ND) transplant-eligible patients. In PART 1 patients were randomized (1:1) in order to receive VTd or D-VTd induction/consolidation; in PART 2, all patients who gained ≥PR after consolidation underwent a second randomization (1:1) in order to receive DARA maintenance or observation only (OBS). The induction schedule for each scheme consisted of four 28-day cycles. In the D-VTd arm, DARA was administered intravenously (IV) at a dose of 16 mg/kg of body weight once weekly in induction cycles 1 and 2 and once every 2 weeks both in induction cycles 3 and 4 and during consolidation (after ASCT). The primary end-point of the study was the rate of sCR after consolidation (day +100 post-ASCT). MRD negativity evaluated at the same time point counted as a secondary study target. The rate of sCR resulted higher in the experimental arm (28.9% vs. 20.3%) and all study subgroups obtained deeper responses with D-VTd regimen (including patients with unfavorable cytogenetics or ISS disease stage III). Likewise, NGS analysis (10^−5^) revealed a higher MRD negativity rate in the D-VTd group, both for patients who achieved ≥CR (33.7% vs. 19.9%) and ≥VGPR (62.2% vs. 42.6%). Median PFS and OS were not reached in either group but achievement of sCR and MRD negativity was associated with a longer time-to-event of disease progression or death [21]. Thus, in a forward-looking way, PART 1 demonstrated the validity of MRD negativity status as a predictor of superior outcomes and as a surrogate endpoint of PFS and OS. 

Phase II **GRIFFIN** RCT investigated the efficacy of another quadruplet, D-RVd, in TE patients with NDMM [22]. After induction with D-RVd followed by ASCT, the experimental arm received two 21-day-cycles of D-RVd consolidation, with Daratumumab administered at a dose of 16 mg/kg every 2 weeks, and up to 26 cycles of LENA-DARA maintenance. The primary end-point was the sCR rate after consolidation. Following protocol amendment 2, MRD was evaluated at four different moments: after induction, after consolidation, and after 12 and 24 months of maintenance. ORR evaluation demonstrated higher sCR rates in the D-RVd cohort both after ASCT-consolidation (42.4% vs. 32%) and after a median FU of 22.1 months (62.6% vs. 45.4%). These results were confirmed in all study subcategories except for high-risk patients. Furthermore, the D-RVd cohort obtained a superior MRD negativity rate at all time points: 21.2% vs. 5.8% by the end of induction, 47.1% vs. 16.5% by the end of consolidation, and 51% vs. 20.4% after a median FU of 22.1 months. These data showed that Daratumumab deepens responses which continue to improve over time. Even high-risk patients obtained higher MRD negativity but the results were not statistically significant. Lastly, PFS and OS were not reached in either cohort. Hematologic adverse events and infections were more common with D-RVd but none of them led to death [23].

The phase II **GMMG-CONCEPT** trial investigated the quadruplet combination isatuximab-carfilzomib-lenalidomide-dexamethasone (Isa-KRd) in patients with high-risk (HR) NDMM. Data obtained after a median follow-up of 24.9 months have shown a 1 year-PFS of 79.6% while the median 2 years-PFS was not reached [24].

Lastly, the **MASTER** trial is a phase II trial that showed that patients characterized by 0 or 1 HRCAs who obtain reiterated MRD negativity with D-KRd can enter FU rather than continuative maintenance therapy. More specifically, the study population received 4 28-day cycles of D-KRd induction, ASCT, and 0, 4, or 8 28-day cycles of D-KRd consolidation according to MRD status (<10^−5^). MRD was assessed at different time points: post-induction, post-ASCT, and every 4 cycles of consolidation. Achievement of MRD negativity represented the primary endpoint and if patients resulted in MRD negative after 2 consecutive evaluations, treatment could be suspended in favor of therapy-free surveillance. On the other side, MRD-positive patients after consolidation underwent LENA maintenance. After a median FU of 25.1 months, 42% of patients were MRD negative after induction, 73% after ASCT, and 82% during consolidation. MRD resurgence or progression after 12 months during therapy-free surveillance occurred in 27% of patients with >2 high-risk cytogenetic abnormalities (HRCAs), while patients with 0 or 1 HRCA experienced a considerably lower rate of these events (0 and 4% respectively) and obtained longer PFS (91 and 97% vs. 58%) [25]. 

At present, the only parameter related to OS is MRD. Thus, it is necessary to standardize MRD and assess it with a reproducible methodology, in reference laboratories and at specific time points. This would allow us to measure the depth of response in an accurate way and proceed with MRD-driven therapy. Furthermore, it would be convenient to assess MRD after induction therapy and at day +100 post-ASCT (according to CASSIOPEIA). The persistence or the absence of MRD positivity after the first ASCT could be employed as a screening marker to select those patients who should start the maintenance therapy (MRD negative) and those who need to undergo a second ASCT.

#### 3.2.2. Transplant Ineligible Patients

MM is a disease of older adults, reflected by a median age at diagnosis of 69 years; 1/3 of these patients is 75 years old and until a few years ago elderly patients were not even included in clinical trials. In order to identify those not-transplant-eligible (NTE) patients with a major probability of death, discontinuation of therapy, and hematologic toxicity, we employ several frailty scores (FS) [26]. 

In 2015 the International Myeloma Working Group (IMWG) developed a geriatric assessment (GA) based frailty index (IMWG-FI) which includes 4 parameters (age, Charlson Comorbidity Index, CCI, Katz Activity of Daily Living, ADL, Lawton Instrumental Activity of Daily Living, IADL), and categorizes patients in 3 groups: fit (score 0), intermediate fit (score 1) and frail (score ≥ 2) [27].

An alternative scoring system is the Revised Myeloma Comorbidity Index (R-MCI) which includes other parameters namely renal/lung impairment, Karnofsky Performance Status (KPS), and high-risk cytogenetic aberrations (HRCAs). According to the score, a patient can be identified as fit (score ≤ 3), intermediate fit (score 4–6), and frail (score > 6) [28].

The Simplified Frailty Scale (SFS) is based on age, CCI, and European Cooperative Oncology Group performance status (ECOG-PS). It was employed in NTE NDMM patients in the phase III FIRST trial and distinguishes frail from not frail patients [29].

All these scales not only serve to guide the clinician in treatment choice but also have strong prognostic value in terms of treatment response, PFS, and OS and must then be integrated with classic staging systems. 

The International Staging System (ISS), the Revised-ISS (R-ISS), and the second revision of the ISS (R2-ISS) are widely used for risk stratification. ISS simply combines serum albumin and β2-microglobulin values while R-ISS includes lactate dehydrogenase (LDH) and cytogenetic abnormalities [del(17p), t(4;14), t(14;16)]. Both systems stratify patients into 3 stages with different survival. Lastly, R2-ISS replaces t(14;16) with the amplification of 1q21 and stratifies patients as low risk/low-intermediate risk/intermediate-high risk/high risk, with different OS and PFS [30]. Other factors that predict un unfavorable prognosis are the presence of CTPCs and extramedullary disease [30]. 

The various frailty scores and staging systems are summarized in Table 1 and Table 2 respectively.

The 3 therapeutic regimens predominantly employed in fragile not transplant eligible (NTE) patients are daratumumab-lenalidomide dexamethasone (D-Rd), daratumumab-melphalan-dexamethasone (D-VMP) and bortezomib-lenalidomide-dexamethasone (V-Rd) [20]. Hereinafter we describe the 3 pivotal studies that led to the approval of the aforementioned regimens in this category of patients. We also discuss the importance of some other potentially employable schemes.

The multicentre, randomized, phase III **MAIA** trial enrolled 737 NTE patients in order to compare the efficacy of the lenalidomide-dexamethasone scheme (Rd) without and with daratumumab (D-Rd). Half of the population was described as frail according to SFS (median age 73 years, ECOG 0–2, HRCAs 15%). Patients with a higher score had a shorter duration of response. However, all patients included in the D-Rd cohort had a better prognosis in terms of PFS, OS (both NR after a median FU of 56 months), and ORR (51% ≥ CRs, 30% VGPRs vs. 30% and 27% respectively) and experienced acceptable toxicity [31].

The open-label, phase III RCT **ALCYONE** studied the efficacy of the bortezomib-melphalan-prednisone combination (VMP) without and with daratumumab (D-VMP) in 706 NTE patients [350 in D-VMP arm, 356 in VMP one, median age 71 y, ECOG 0–2, HRCAs 14% (98 patients)]. Data obtained after 36 months of FU have shown that the experimental arm achieved superior PFS (51% vs. 18%), OS (78% vs. 68%) and ORR (46% ≥ CRs and 27% VGPRs vs. 25% and 24% respectively) [32]. In both MAIA and ALCYONE trials NGS (10^−5^) was employed to assess MRD status and 1/3 of the population treated with D-Rd and D-VMP reached MRD negativity and achieved the longest PFS.

The open-label, phase III RCT **SWOG S0777** investigated the efficacy of the Rd scheme without and with bortezomib (V-Rd) in previously untreated NTE patients (median age 65y, ECOG 0–3). The study population was randomized according to ISS and intention to transplant (yes vs. no). After 7 years of FU, the experimental arm showed superior results in regard to PFS (41 vs. 29 months), OS (NR vs. 69 months), and ORR (24% ≥ CRs and 51% VGPRs vs. 12% e 41% in Rd arm). The advantage obtained in terms of OS was independent of age and thus the scheme could be used even in >75 years old patients. However, the ORR and PFS obtained with V-Rd are lower than with DARA-based regimens [33].

The open-label, phase III RCT **ENDURANCE** investigated the employment of the Rd scheme in association with proteosome inhibitors [carfilzomib (K) and bortezomib (V)] as a first-line treatment in patients with NDMM who were not planned for immediate ASCT (median age 65y, 31% ≥ 70 y). After a median FU of 9 months, the study proved that there was no significant difference in terms of PFS and OS between VRd and KRd (median PFS was 31.7 vs. 32.8 months respectively, while median OS was not reached) [34].

The **HOVON-143** trial (NTR6297) is a prospective, multicentre phase II trial conducted in 39 hospitals throughout the Netherlands and Belgium which investigated the ixazomib-daratumumab-dexamethasone (IDd) scheme in a population of 65 frail patients (IMWG-FI). IDd was administered for 9 cycles followed by 2 years of ID maintenance. After a median FU of 22.9 months, the median PFS was 13.8 months, the 12-month OS rate was 78% and the ORR was 78% (CRs 8%). Patients experienced an improvement in the quality of life (QoL) after only 3 cycles and developed low-grade toxicity. Furthermore, considering that 80 years old patients without comorbidities managed to achieve a 22 months PFS, the study proved that age alone is not a major predictor of unfavorable prognosis [35].

Lastly, the phase III RCT **TOURMALINE-MM2** studied the efficacy of the Rd scheme without or with ixazomib [IRd (ixazomib 4 mg for 18 cycles and then 3 mg as maintenance therapy)]. However, the trial did not meet the threshold for statistical significance and therefore the primary end-point of PFS was not met. On the other hand, the study did not entail any OS advantage compared to standard Rd (NR in both arms) [36].

At present, there are very few real-life studies aiming to investigate the use of >2 drug combinations on an NTE population. 

### 3.3. Excursus on Treatment: Relapse

#### 3.3.1. First Relapse

MM is a chronic disease that tends to further relapse with a progressively shorter time to the next treatment (TNT). For this reason, it is of crucial importance to achieve the deepest response by accurately choosing the II-line therapy. More specifically, in young fit patients the primary aim is to reach CR with MRD negativity, in old fit/young patients with comorbidities, the goal is to increase survival with a good QoL, and lastly, in old unfit patients, the intent is the chronicity of the disease and the prevention of drug toxicity [37].

The choice of salvage therapy depends on several factors: ISS and R-ISS stage, high cytogenetic risk, aggressive clinical presentation [plasma cell leukemia (PCL); extramedullary disease (EMD)], functional high risk (primary refractory disease; early relapse after ASCT), host factors (age, ADL, IADL, CCI, and previous toxicities), features at the time of relapse (biochemical vs. clinical) [38].

According to IMWG 2021 guidelines, primary refractory patients can be distinguished according to their refractoriness or not to lenalidomide (LENA) [20]. Refractoriness is defined as no response to lenalidomide or progression within 60 days of the last dose.

In patients not refractory to lenalidomide, in recent years, 4 phase III trials have contributed to the approval of 4 new lenalidomide-based triplet combinations in which Rd regimen is associated with proteosome inhibitors [like carfilzomib (K-Rd) in the **ASPIRE** trial [39] and ixazomib (Ixa-Rd) in the **TOURMALINE-MM1** trial [36]] or monoclonal antibodies [like daratumumab (D-Rd) in the **POLLUX** trial [40] and elotuzumab (Elo-Rd) in the **ELOQUENT-2** trial [41]]. All these triplets have demonstrated a benefit over Rd doublet. By comparing these trials in a hazard ratio-based-meta-analysis, we can realize that the triplet D-Rd shows the lowest PFS and OS hazard ratio. However, in high-risk patients, these data are not substantially different from those obtained from K-Rd and Ixa-Rd [42].

Only 12–20% of patients included in the studies were >75 years old. According to the POLLUX trial, the median PFS of this subcategory resulted lower than the entire population (28.9 vs. 44.5 months in the D-Rd arm) [40], while in the ASPIRE trial, it was comparable (30.3 vs. 26.3 months) [39]. Furthermore, the older population presented a higher rate of AEs (hematologic toxicity).

On the other side, clinical studies on LENA-sparing regimens initially included only a limited number of patients previously exposed to lenalidomide. For example, in the CASTOR trial [a phase III study on the daratumumab-bortezomib-dexamethasone combination (D-Vd)] LENA-refractory patients were just 50% of the entire population [43].

On the contrary, the phase III RCTs **IKEMA** [44] and **CANDO**R [45] (the former comparing Isa-Kd vs. Kd and the latter comparing D-Kd vs. Kd) recruited a higher number of patients previously exposed to lenalidomide (197/466 in CANDOR, 143/302 in IKEMA) who were both primary and multi refractory. Lastly, in the phase III trial **OPTIMISMM**, which evaluated the efficacy of the combination pomalidomide-bortezomib-dexamethasone (PVd), all the study population had already been exposed to lenalidomide [46]. 

None of these trials, however, tried to investigate the difference between LENA-refractory and non-refractory populations in terms of PFS. 

A hazard ratio-based network meta-analysis revealed that the best drug combination in LENA-refractory/exposed patients should include an anti-CD38 and a PI and that D-Vd (or D-Kd/Isa-Kd when available) has the highest probability of being the best treatment in this setting [38]. According to the IKEMA trial, Isa-Kd is the only regimen that can reach 3y of PFS in patients with R/R-MM [44]. On the other hand, the CANDOR trial showed that D-Kd can achieve 28 months of PFS. From these data, it might seem that Isa-Kd is superior to D-Kd (38). However, the efficacy of these schemes is fully comparable in terms of hazard ratio [38].

Lastly, the P-Vd scheme (OPTIMISMM) has proved to be advantageous in both LENA-refractory and LENA-exposed patients, obtaining a PFS of respectively 18 and 22 months. Unfortunately, there aren’t current real-life data on the use of P-Vd instead of Isa-Kd [46].

Real-world analysis has shown that nearly 60% of patients are ineligible for phase III trials mentioned above due to comorbidities and the subsequently increased risk of toxicity. Therefore, it is clear that it is not always possible to abide by the guidelines derived from protocols since there is a percentage of patients who cannot benefit from certain therapeutic schemes.

With regard to II-line therapies, mention should be made of salvage ASCT. Prior to the introduction of maintenance therapy with lenalidomide, the appropriate remission duration to assess the possibility of a second ASCT was 18 months from the first transplant. Thanks to maintenance therapy it was possible to achieve a median disease-free remission of 50–60 months [47], following which the IMWG guidelines were modified by setting at 36 months the remission duration necessary to evaluate salvage ASCT [48]. 

A phase III German trial compared rescue ASCT + lenalidomide maintenance to continuative therapy with Rd regimen and showed that receiving a second transplant improves PFS (23 vs. 20 months) and OS (NR vs. 57 months) while there is no difference in response rates [49]. Moreover, in the last retrospective study of the German group, re-induction with K-Rd followed by rescue transplant determined a PFS comparable to that obtained after the first transplant [50]. Lastly, the K-Rd regimen has also been investigated as maintenance therapy in the phase II **FORTE** trial [51].

##### Real-Life 

Data from real-life experiences have widely confirmed the efficacy of daratumumab-based regimens and K-Rd in first relapse [52,53,54,55,56].

Regarding the K-Rd regimen, Conticello et al. investigated the feasibility and tolerability of this combination in 130 R/R-MM patients (95 with 1–2 prior LOTS, 19 with HRCAs; 32% with ISS III). The median PFS was 22.9 months, median OS was NR. Creatinine clearance > 30 mL/min, quality of the best-achieved response, and standard fluorescence in situ hybridization (FISH) risk were independent predictors of a favorable outcome. Patients who received the full-dosage of carfilzomib in the first 2 cycles had a better outcome [52].

A retrospective survey held at 14 different institutions from Southern Italy evaluated the efficacy of K-Rd in 123 patients with a median of 2 previous LOTs (range 1–9) and a median age of 63 years (range 39–82). At the time of analysis, median number of courses administered was 11 (range 1–34). ORR (CR+VGPR+PR) was 85%. After a median FU of 27 months, mOS and mPFS were 33 and 23 months, respectively [53].

A real-life study on K-Rd carried on by Mele et al. obtained significant results. It included 130 patients [ISS III 42%, 8 with HRCAs; median LOTs 1 (1–11)] treated between December 2015 and August 2018. The ORR was 79% and 2y-PFS/OS was 54 and 70% respectively. Median PFS was longer (32.4 months) in patients achieving a VGPR. Twenty-one patients performed ASCT after KRD achieving a 2y-PFS of 100%. Accordingly, K-Rd should be considered as a bridge regimen to ASCT. Cardiovascular events occurred in 11% of patients [54].

Antonioli et al. investigated the D-Rd regimen in 44 R/R-MM patients (ISS III 24%; HRCAs 34%; 1 prior LOT 71%). After 1 year of FU, PFS was 60% and OS 81% [55]. 

Lastly, A report from The MM Gimema Lazio group analyzed the efficacy of D-Rd vs. D-Vd in 171 R/R-MM patients (120 were primary refractory). The study showed that mOS was not significantly different between the 2 arms. On the contrary, ORR was 93% in the D-Rd arm and 76% in the D-Vd arm (CR+VGPR 81% vs. 49% respectively) [56].

All these data are summarized in Table 3.

#### 3.3.2. Beyond the First Relapse 

Multi-refractory patients have a drastically shorter survival. In this regard, it is of primary importance to evaluate all the clinical and laboratory features and to analyze in detail the response and tolerability to antecedent therapies to screen patients for the most appropriate treatment.

Combining mAbs with pomalidomide or carfilzomib could be the winning strategy. The phase III RCT **APOLLO** analyzed the effect of daratumumab-pomalidomide-dexamethasone (D-Pd) vs. Pd alone in patients with HRCAs, refractory to prior LOTs (including LENA and a PI). Data obtained from the experimental arm after 16 months of FU confirmed a median PFS of 12.4 months in 52% of cases, a median ORR of 69% (9% sCR, 15% CR, 26% VGPR and 18% PR) and a 9% MRD negativity rate [57].

The phase II **ELOQUENT** trial focused on the elotuzumab-pomalidomide-dexamethasone combination (Elo-Pd) in patients with R/R-MM and ≥2 LOTs (including LENA and a PI). The scheme achieved 10.3 months of PFS (vs. 4.7 months in the Pd arm), a 41% reduction in the risk of death, and a 1y increase in median OS [58]. 

The phase III **ICARIA-MM** study analyzed the efficacy of the Pd regimen without and with isatuximab (Isa-Pd). It included patients who had at least 2 previous LOTs (including LENA and a PI) and a glomerular filtration rate (eGFR) < 60 mL/min. LENA-refractory patients were 94%. Isa-Pd association managed to reach a median PFS of 11 months (vs. 5.9 months), a median OS of 24.6 months (vs. 17.7 months), an ORR of 63% (vs. 33.3%) and a longer TTNT (15.5 months vs. 8.9). Furthermore, time from randomization to progressive disease (PD) on subsequent therapy or death (PFS2) was longer in the experimental arm than in the control one (17.51 vs. 12.88 months). Neutropenia rates were lower with Isa-Pd and episodes of grade 3 respiratory infections were not frequent (3.3%) [59].

Two of the studies mentioned in the previous paragraph, **IKEMA,** and **CANDOR**, also included patients with more than one prior LOT. More specifically, IKEMA (Isa-Kd vs. Kd) included patients with 1–3 prior LOTs (e.g., bortezomib and LENA), a small percentage of patients aged >75 y (mainly in the Isa-Kd arm) and high-risk patients with 1q21. PFS at 18 months for Isa-Kd vs. Kd was: 77% vs. 64%, 1 prior line; 68% vs. 45%, >1 prior line; 53% vs. 31%, lenalidomide-refractory patients; and 63% vs. 43%, bortezomib-refractory. Moreover, higher ≥VGPR and MRD negativity rates were observed across all subgroups of the experimental arm [60]. The other study, CANDOR (D-Kd vs. Kd), showed the superiority of D-Kd even in those patients who have already received ≥2 LOTs or autologous SCT [45].

The phase II **DREAMM** study is the reference study for the antibody–drug conjugate (ADC) belantamab mafodotin. More specifically, belantamab is a BCMA inhibitor conjugated to the cytotoxic microtubule inhibitor monomethyl auristatin F (MMAF), indicated in those patients who have already undergone 4 LOTs and are refractory to the 3 principal classes of drugs (i.e., PI, IMiD, and anti-CD38). In the study, patients were randomized in two cohorts according to HRCAs and the number of prior LOTs (≤4 vs. >4). In the first cohort, belantamab was administered at a dose of 2.5 mg/kg, while in the second cohort, the dosage was 3.4 mg/kg. The primary endpoints of the study were ORR and safety. The ORR was similar in both cohorts (31% vs. 34%) with a median duration of response (DoR) of 11 months and a median OS of 13 months. Median PFS was 2.8 months in the 2.5 mg/kg cohort and 3.9 months in the 3.4 mg/kg cohort. With regard to safety, the study showed that the most common TEAE (in both cohorts) was keratopathy [61] which is actually reversible with discontinuation of therapy or dose reduction [62]. Condorelli et al. have reported a real-life experience with heavily pre-treated patients who received belantamab, confirming the efficacy and safety profile of this new drug [63]. 

As of 31 March 2022, the DREAMM-2 trial median follow-up was 12.48 and 13.77 months for patients randomized to 2.5 mg/kg (N = 97) and 3.4 mg/kg (N = 99), respectively. Data obtained from this analysis confirmed that one-third of patients responded to this therapy (ORR was 32% and 35%, respectively); 19% and 24% of patients achieved VGPR with a 36% and 23% MRD negativity rate. Interestingly, the median duration of response was 1 year in the lower dose cohort and just 6.2 months in the 3.4 mg/kg cohort. Median OS improved remarkably, reaching 15.3 and 14.0 months. Final data from this FU also confirmed that ocular events are generally transient (resolution in nearly 86% of cases) and only 3% of patients in both cohorts discontinued treatment. The most commonly reported ocular events were: keratopathy (71%), blurred vision (23%), best corrected visual activity (BCVA) reduced to 20/50 (21%), and dry eye (15%); no permanent complete vision loss occurred. The data were presented at the American Society of Hematology annual meeting (ASH 2022) held in New Orleans [64].

Two novel drugs that represent a potential novel treatment to employ when myeloma is refractory to current therapeutic options are: selinexor, a selective inhibitor of exportin 1 (XPO1) [phase 2b STORM trial [65], and Iberdomide, a new IMiD whose mechanism of action consists in the degradation of proteins responsible for resistance (Ikaros and Aiolos) [phase I/II CC-220-MM-001 trial [66]].

Selinexor represents a new, non-cross-resistant therapy with a novel mechanism of action. The **STORM** trial enrolled 122 patients with a median of 7 prior LOTs and 53% of them had HRCAs. They received oral selinexor (80 mg) plus dexamethasone (20 mg) twice weekly. The primary end point was ORR and 26% of patients obtained a PR or better (2 sCR). Median DoR, PFS, and OS were 4.4 months, 3.7 months, and 8.6 months respectively. Gastrointestinal toxicity (nausea, vomiting, and diarrhea) represented the major AE determined by this drug. Thrombocytopenia occurred in 73% of patients and 6 of them had bleeding events of grade 3 or higher [65]. Based on these results Selinexor was approved in the US for the treatment of penta-refractory patients.

Cornell et al. analyzed the efficacy of the selinexor-dexamethasone (sel-dex) combination in comparison with other common regimens employed in multi-refractory patients. More in detail, the analysis included 64/122 patients from STORM, treated with sel-dex, and 125/278 patients from the retrospective **MAMMOTH** study (Monoclonal Antibodies in Multiple Myeloma: Outcomes after Therapy failure), who received MM-therapy other than sel-dex (mostly pomalidomide-based regimens and traditional chemotherapy). All these patients were penta-refractory and triple-class-refractory (TCR). Patients in STORM had better OS (10.4 months) and ORR (32.8%) than patients in MAMMOTH (6.9 months and 25.0% respectively). Results from this analysis demonstrated that the employment of non-cross-resistant drugs such as selinexor allows the treatment of even those patients who are refractory to novel agents such as anti-CD38 antibodies. Despite the regimens used in MAMMOTH were less effective, it should be specified that they all represented agents that these patients had previously received. This likely reflects the reuse of these agents combined with other agents as part of alternative regimens [67]. 

Moreover, several studies have been made in an attempt to combine selinexor with other drugs that can potentiate its action. The phase III **BOSTON** study compared the efficacy of the combination selinexor-bortezomib-dexamethasone (SVd) vs. bortezomib-dexamethasone (Vd) in patients exposed to 1–3 prior LOTs. After a median FU of 13.2 months, the median PFS resulted higher in the SVd arm (13.9 months vs. 9.5) [68]. Lastly, the **STOMP** study is an ongoing phase Ib/II study that has demonstrated encouraging efficacy and safety with once-weekly sel-dex in combination with common backbone therapy agents such as pomalidomide, lenalidomide, daratumumab, bortezomib, or carfilzomib [69].

As mentioned above, the other novel drug is Iberdomide. The phase I/II **CC-220-MM-001** trial investigated the efficacy of iberdomide-dexamethasone (Id) combination. The study population was divided into two cohorts: in the first (the largest one) there were those refractories to IMiDs, PIs, and anti-CD38, and in the second those previously exposed to anti-BCMAs. The first cohort also included 25–30% of patients with extramedullary disease. Both cohorts received iberdomide at doses of 1.6 mg, obtaining disease control in 80% of cases, median duration of response of 30.3 months with an OS of 46 months. Hematological toxicity was easily manageable. An interesting aspect is that it was observed an increase in proliferating NK cells and T cells during treatment. Currently, there are studies that plan to associate this scheme with anti-CD38 mAbs [66].

##### Real-Life

**LocoMMotion** is the first prospective study of real-life SOCs in triple-class exposed patients with R/R-MM. Patients [N = 248; ECOG PS of 0–1; ≥3 prior LOTs (IMiD, PI, anti-CD38 mAb) or double refractory to a PI and IMiD] were treated with median 4.0 (range, 1–20) cycles of SOC therapy. Overall response rate was 29.8%. Median progression-free survival (PFS) and median overall survival (OS) were 4.6 and 12.4 months. Death occurred in 107 patients due to PD. The 92 varied regimens utilized demonstrate a lack of clear SOC for heavily pretreated, triple-class exposed patients with RRMM in real-world practice. As a result, outcomes are generally poor [1]. 

An Italian multicenter experience evaluated the efficacy of Elo-Rd vs. KRd in poli-refractory patients (median age 67 y; 47% with 2 prior LOTs; 33% LENA-exposed). After a median FU of 18 months, both triplets obtained comparable results in terms of PFS and OS. In univariate analysis, ISS 3, previous LENA exposure, age >65 y and number of prior lines were predictors of worse prognosis [70].

Markovic et al. investigated the Dara-single agent in a retrospective real-life survey of 44 RR patients treated at 10 different centers in southern Italy. Probands had already received a median of 4 previous LOTs and 60% of them were characterized by HRCAs. Median FU was 7.8 months; median PFS and OS were 7.2 and 7.8 months respectively. Those who obtained VGPR and CR (27%) achieved a PFS of 29.5 months and an OS of 30.6 months. Furthermore, PFS was longer in those previously treated with pomalidomide-dexamethasone (9.3 vs. 3.4 months) rather than with K-Rd. In both univariate and multivariate analysis, PFS and OS resulted in superior if patients obtained PR or better within 6 months [71]. 

Regarding Elo-Pd, an Austrian-German study analyzed a cohort of 22 patients with a median of 5 LOTs (including pomalidomide) who received a median of 5 Elo-Pd cycles. Low tumor burden was associated with improved PFS (13.5 months for patients with ISS stage I/II at study entry vs. 6.4 months for ISS III). The ORR was 50% and objective responses were also seen in 5 patients who had been pretreated with pomalidomide [72].

All the real-life studies of patients with ≥2 LOTs presented in this section are summarized in Table 4.

#### 3.3.3. T-Cell-Directed Immune Therapy

Triple and penta-refractory patients have a median survival of less than a year. The introduction of bi-specific antibodies and chimeric antigen receptor (CAR) T-cell therapy in the therapeutic arena allows to treat and potentially cure this difficult category of patients. 

Bi-specific antibodies (BsAbs) represent a new class of drugs engineered to bind both a target on neoplastic PCs and on cytotoxic immune effector cells. By this immunologic synapse, they lead to T-cell activation and destruction of MM cells. More specifically, they target the B-cell maturation antigen [BCMA (teclistamab, elranatamab)], the orphan G-protein-coupled receptor, class C group 5 member [GPRC5D (talquetamab)] and Fc receptor homologous 5 [FcRH5 antigen (cevostamab)] expressed on MM cells and CD3 receptor expressed on T-cells. A difference between BCMA and GPRC5D antigens is that the former is primarily present on B precursors and PCs, while GPRC5D is also found on keratinized structures such as hair, nails, and tongue. Preclinical studies are also investigating NK-cell engagers as a novel mechanism of action. BsAbs possess the fragment crystallizable region (Fc region) and for this reason, they have more stability and longer half-life than Bi-specific T-cell engagers (BiTEs). 

On the other hand, CAR-T cell therapy represents a novel form of immunotherapy which implies the use of T cells collected through apheresis from patient’s PB and genetically re-engineered in order to express surface proteins called chimeric antigen receptors (CARs). Thanks to this structure CAR-T cells can recognize and bind to specific surface antigens of malignant cells (e.g., BCMA (Ide-cel, Cilta-cel) and GPRC5D (MCARH109)) unleashing their anti-neoplastic activity. CAR-T cells are also able to increase memory T-lymphocytes, prolonging their effect and persistence in vivo. Unfortunately, the majority of patients who receive CAR-T cell therapy tend to relapse due to a mechanism of T-cell exhaustion, increase of T-regulatory cells, or down-modulation of the target antigen caused by tumor cells.

The most remarkable side effect caused by both BsAbs and CAR-T cells is cytokine release syndrome (CRS). The most severe form (grade 3–4), which involves hospitalization in intensive care, does not have a very high incidence. The same applies to neurotoxicity, which represents another possible side effect [73].

#### 3.3.4. Chimeric Antigen Receptor T Cell Therapy

There are several CAR-T preparations in trials, but at the moment the best results seem to be from the phase 1b/2 **CARTITUDE-1** study, which evaluated the safety and efficacy of Ciltacabtagene autoleucel (Cilta-cel). This is an anti-BCMA CAR-T cell therapy tested on double-refractory patients treated with 3 or more lines of therapy including a PI, IMiDs, and an anti-CD38. A total of 97 patients were enrolled, 1/4 of whom had HRCAs; 13% of them had extra-medullary disease, 40% were penta-refractory, and 85% were triple-class-refractory. The median time from diagnosis to CAR-T treatment was 6 years (ranging from 1.6 to 18 years); the primary endpoint was ORR. At a median follow-up of 27.7 months, an overall response rate of 97.9% with a stringent complete response rate of 82.5% was recorded. PFS and OS were 54.9% and 70.4% respectively; 61 patients were evaluated for MRD in NGS and most achieved MRD negativity (10^−5^). In those who had sustained MRD (>6 months) PFS at 27 months was 73%. CRS occurred in most cases (55% of them were grade (G) 1–2 and required Tocilizumab) but only 1% was G4. Sixteen patients (20% of the population) developed immune effector cell-associated neurotoxicity syndrome (ICANS), 12 had extra ICANS neurotoxicity, and 8 had ICANS first and then other forms of neurotoxicity [74].

The multicohort phase II **CARTITUDE 2** study investigated the use of Cilta-cel in a wide variety of MM-affected patients, including newly diagnosed (cohort E and F) and patients who had already been treated with an anti-BCMA (belantamab or BsAbs) (cohort C). Considering the excellent results given by Cilta-cel, the achievement of MRD negativity was established as the primary endpoint. Data extrapolated from the study showed that patients in cohorts E and F obtained an ORR of about 100%, and 90% of them achieved MRD negativity (10^−5^), while cohort C reached a 62% ORR and only 5 patients resulted as MRD negative. It was therefore found that patients already treated with an anti-BCMA, in particular those who have received it as a line of therapy immediately prior to CAR-T infusion, had a lower response to Cilta-cel and shorter DoT [75]. 

Idecabtagene vicleucel (Ide-cel) is another anti-BCMA CAR-T cell therapy that has been investigated by the phase II **KarMMA** trial in poly-refractory patients (≥3 LOTs with ≥2 consecutive cycles each) or refractory to last prior therapy according to IMWG. The study enrolled 140 patients of whom 128 received Ide-cel infusion. The primary endpoint was ORR. After 13.3 months of FU, the ORR was 73% (94/128), 42 patients (33%) obtained a sCR (median time to response was 2.8 months) and 33 of them (79%) had indetectable MRD (<10^−5^). A total of 67 patients (52%) had a very good partial response or better. Median DoR and PFS were higher in patients receiving CAR-T at the dose of 450 × 10^6^ (11.3 and 12.1 months respectively) and in those achieving ≥CR (19.0 and 20.2 months). The median OS was 19.4 months. Ide-cel proved to be beneficial in all study subcategories (older patients, aggressive disease, HRCAs, triple- or penta-refractory disease, high tumor burden, and extramedullary disease). CRS occurred in 107 patients (7 with grade 3 or higher) while ICANS occurred in 23 patients who were ≤grade 3. Twenty-eight patients experienced a disease progression and were retreated with Ide-cel at a higher dose, reaching a second response with a duration of 1.9–6.8 months [76]. 

The retrospective real-world study **KarMMA-RW** compared 190 RR patients (Eligible RR Cohort), treated with conventional therapeutic regimens, with 128 patients from the KarMMA trial. The 3 most employed regimens were KP-d, Elo-Rd, and KC-d. After 13.3 months of FU, the ORR, PFS, and OS were significantly higher in the KarMMA cohort rather than the Eligible one (76.4% vs. 32.2%, 11.6 months vs. 3.5 months, and 20.2 months vs. 14.7 months respectively) [77].

Most recently, a phase III study compared the administration of Idecabtagene vicleucel vs. different standard therapy regimens (mainly Dara-Pd, Kd, Elo-Pd) in patients with relapsed/refractory diseases who had received two to four previous therapy regimens and with disease refractory to the last therapy. A total of 386 patients were included in the study, 66% of patients were triple refractory and 95% were refractory to daratumumab. A response was observed in 71% of patients treated with ide-cel vs. 42% of patients treated with conventional therapy. After a median of 18.6 months, PFS was 13.3 months in the Ide-cel group vs. 4.4 months in the standard therapy group. Although the results of using Ide-cel seem inferior to those obtained with Cilta-cel, this study represents definitive confirmation that the use of CAR-Ts is the most effective therapy for triple-refractory patients [78]. 

Recently, a phase I, first-in-class clinical trial reported that 7/10 patients who relapsed after BCMA-directed CAR-T therapy responded to GPRC5D-directed CAR-T MCARH109 [79].

#### 3.3.5. Bispecific Antibodies and the Importance of Sequencing in the Era of Immunotherapy

Bispecific antibodies represent an off-the-shelf therapy that can be extremely beneficial for those heavily pretreated patients with rapidly progressive disease that should be promptly treated and can’t wait for lymphocyte apheresis and CAR-T manufacturing. Furthermore, BsAbs appear as a very effective strategy in those patients who relapse after CAR-T cell therapy. On the contrary, employing CAR-T cells after BsABs (CARTITUDE-2, cohort C) or retreating patients with the same CAR-T product (KarMMA-2, subgroup analysis) seems less effective. At the moment, it is not possible to define an evidenced-based sequencing strategy. However, data from phase I and II trials allowed us to understand that both addressing the same target (e.g., BCMA) or changing it (e.g., GPRC5D) produces encouraging results.

The phase II **MajesTEC** study focused on teclistamab which is a humanized IgG4, anti-BCMA BsAb. A total of 165 patients was enrolled and had a median of 5 prior therapies (range from 2 to 14); 30% were penta-refractory (after two IMiDs, 2 PIs, and one anti-CD38); one-quarter of them had HRCAs; and one-fifth had an extra-medullary disease. After a median FU of 14 months, the ORR was 63% (40% ≥ CRs, 19.4% VGPRs, 4.2% PRs) and a quarter of patients also achieved MRD negativity (as measured by NGS); median response time was about 1.2 months, while median PFS was nearly a year. With regard to toxicity [73], although cytokines release syndrome (CRS) occurred in 70% of cases and neurotoxicity in 14%, the advanced form of either occurred in only one patient, respectively. The study also included those patients who had been previously treated with anti-BCMA therapy (ADC or CAR-T immunotherapy) (cohort C). These patients achieved a promising response in more than 50% of cases. As a consequence, anti-BCMA BsAbs represent a valid option in those patients who relapsed after anti-BCMA CAR-T therapy [80].

The Phase 1 first-in-human study **MagnetismMM-1** evaluates the safety, pharmacokinetics (PK), pharmacodynamics, and efficacy of elrnatamab for RR patients. Elranatamab is humanized IgG2a BCMA-CD3 BsAb which has shown promising results even in those patients previously treated with anti-BCMA strategies. The trial included 55 patients with a median of 5 prior LOTs. Most of the patients (91 %) were TCR, 69% had prior ASCT, and 24% received prior BCMA-targeted therapy. After 1 year of FU, the ORR was 64%, and VGPRs and CRs were 56% and 38% respectively. Moreover, this agent induced durable clinical and molecular responses, and all patients who obtained ≥CR achieved MRD negativity (1 × 10^−5^). Regarding the 13 patients previously treated with anti-BCMA, 46% of them (7/13) achieved ≥VGPR and the median DoR was 17.1 months. As previously said about teclistamab, these results further strengthen the concept that re-addressing the same target with anti-BCMA BsAbs could be an effective strategy to significantly improve the outcome of RR patients. CRS occurred in 67% of patients but was mainly in grade 1 or 2.

The possible association between an anti-CD38 with BsAbs is based on the concept that anti-CD38 mAbs also target regulatory T cells and myeloid suppressor cells which, despite being part of the physiological BM microenvironment, can protect plasma cells from immunological aggressions and induce resistance. In this respect, it should be mentioned the open-label, multicenter, phase III RCT **MagnetisMM-5** which investigates the efficacy and safety of elranatamab (a humanized IgG2a BCMA-CD3 BsAb) without and with daratumumab in MM patients who have received ≥1 previous treatments (including LENA and a PI). The study is currently ongoing in various Italian centers including Catania.

The **MonumenTAL-1** study is a phase I (dose-finding) study which focused on talquetamab, a first-in-class humanized IgG4 anti-GPRC5D BsAbs. Patients were divided into 2 arms: in one the drug was administered at a dose of 405 μgr/kg s.c./week and in the other at a dose of 800 μgr/kg s.c./every 2 weeks. Both doses were well tolerated. CRS occurred in more than 70% of cases (grades 1 and 2) and only one patient had the advanced form (at a dose of 400 μgr/kg). Study data confirmed an ORR of 70% with a median response time of 1 month. Sixteen patients had already been treated with anti-BCMA CAR-T therapy. The ORR of 67% of patients who received Ide-cel and 83% of those who received Cilta-cel was comparable to that of penta-refractory (ORR 78–83%) or TCR patients (ORR 65–70%) who were anti-BCMA naïve [81]. 

New, so-called 2 + 1 bispecific antibodies are alnuctamab [82] and forimtamig. These bispecific antibodies have two possible binding sites (2:1 configuration) and are thus expected to be even more effective. Forimtamig is a GPRC5DxCD3 T-cell-engaging bispecific antibody that has proved to be effective in RRMM with long-term treatment response. Results from a phase 1 dose-escalation study on forimtamig have been recently presented at the ASH annual meeting in 2022. The study included 108 multi-refractory patients, 51 of them were administered forimtamig intravenously (IV), while the other 57 were subcutaneously (SC). The ORR and DoR were 71.4% (≥VGPR: 59.2 %) and 10.8 months in the IV arm, and 63.6% (≥VGPR: 52.8%) and 12.5 months in the SC arm respectively. Furthermore, 18 patients in the study population had already received anti-BCMA therapy and 10 of them achieved a median DoR of 12.9 months (IV) and 8.8 months (SC) [83].

Van Oekelen et al. have analyzed the outcome of 79 patients who relapsed after autologous anti-BCMA CAR-T therapy. Twenty-three of them were treated with anti-GPRC5D and anti Fc receptor-homolog 5 (FcRH5) bispecific antibodies and 6 received salvage anti-GPRC5D CAR-T cell therapy. The ORR in this subgroup of patients was 91.4%. Therefore, the study strongly suggests that targeting alternative myeloma tumor antigens allows to overcome BCMA antigenic loss [84].

For this reason, cevostamab, a bsAb targeting FcRH5 and CD3 is being evaluated in RRMM pts who have received prior anti-BCMA therapy, including anti-BCMA antibody–drug conjugates, CAR T cells, and anti-BCMA bsAbs. Subjects’ enrollment is currently in progress [85].

### 3.4. Out of the Bone Marrow Microenvironment: A Quest for a Therapeutic Algorithm for the Treatment of Extramedullary Disease 

#### 3.4.1. Overview and First-Line Therapy

Extra-medullary myeloma is a spectrum of diverse clinical entities comprising paraosseous (PO), solitary (SP), and extramedullary plasmacytoma (EMP). Focusing on EMP, it represents a tumor mass growing in organs/soft tissue independently from the BM microenvironment. In other words, is the result of the hematogenous spread of clonal PCs. EMD can be present both at diagnosis (0.5–4.8%) or develop in subsequent relapses (3.4–14%) and is characterized by particularly aggressive features (i.e., HRCAs, high Ki67, and drug refractoriness) [86]. EMD response to therapy can be evaluated through PET/CT according to the recently standardized criteria mentioned in previous paragraphs. Unfortunately, an IMWG consensus on both induction and further lines of therapy has not been established yet and there are few prospective trials investigating treatments in EMD. The importance of prospective data is even clearer considering the work of Montefusco et al. who published a meta-analysis of 8 prospective trials including an overall of 2332 NDMM patients. Among them, 243 had a PO and 12 an EMP. Interestingly, treatment with IMiDs (especially lenalidomide) or PIs resulted in a similar PFS and PFS-2 to non-EMD patients who received the same treatment (25.3 vs. 25.2 months). This was the first meta-analysis showing the ability of new drugs to counteract EMD negative prognosis [87]. Previously, this great impact on prognosis could be obtained only with ASCT [88] or double ASCT [89]. A prominent real-world multicenter, retrospective study conducted by Beksac et al. included 130 NDMM EMD patients (92 with EMP) and 96 with RR EMD (84 with EMP). After a median of 2 lines of treatment and ASCT, the ND patient obtained a PFS of 38.9 months and an OS of 46.5 months [90]. 

#### 3.4.2. Trials and Real-Life in Relapsed-Refractory Emd

Relapsed extramedullary disease is characterized by a very dismal prognosis and represents a major unmet clinical need. In the above-mentioned real-world study conducted by Beksac, extramedullary plasmacytoma at relapse showed an estimated PFS of only 9.1 months and an OS of 11.4 months. 

According to evidence in the literature, bortezomib seems to exert a certain effect. Relying on the Mayo Clinic 2017 guidelines [91] and on data from following studies [92], fit patients should be treated with bortezomib-based regimes and consolidated with ASCT (while daratumumab or anthracycline-based regimens should be favored in frail patients). On the other hand, other PIs (carfilzomib; ixazomib) seem to be only partially or minimally beneficial [93,94]. 

Apart from induction therapy, IMiDS are widely used even at the relapse. A retrospective study evaluated the role of LENA-based regimens in 18 RR EMD patients (median 3 prior LOTs). Data obtained showed that the combination LENA-DEX determined an ORR of 61.1% (44.4% disappearance of EMD and 16.6% reduction in size) [95]. Pomalidomide + other drugs (e.g.: bortezomib/ixazomib/daratumumab) proved to be more effective [96] rather than with dexamethasone alone [97]. On the contrary, thalidomide, the first-in-class IMiD, did not show any effect on EMD [98]. 

Despite extramedullary PCs having a lower expression of CD38 receptor, several studies have tried to investigate the effect of anti-CD38 monoclonal antibodies in RR EMD. Isatuximab has shown the most promising effect. The **ICARIA-MM** included 24 patients with EMD, 14 in the Isa-Pd arm and 10 in the Pd arm. Those treated in the experimental arm had a major PFS (4.57 months vs. 1.56) and ORR (50% vs. 10%). Interestingly, daratumumab has shown limited efficacy both in trials [99] and in real life [100]. This difference between isatuximab and daratumumab may be explained by the fact that these two Abs bind different CD38 epitopes. Other drugs such as elotuzumab, selinexor [65], and venetoclax are currently under investigation. 

Furthermore, mention should be made of the role of CAR-T cell therapy in this difficult setting of patients. A study published by the Chinese group on LCAR-B38M included 57 RR patients (17 affected by EMD). After 25.1 months of FU, EMD patients achieved an mPFS of 8.1 months and an mOS of 13.9 months [101]. The same group has recently described the case of a 56-year-old man affected by a penta-refractory EMD who was successfully treated with LCAR-B38M in the **LEGEND-2 study** [102] obtaining a 5-year CR [103]. Deng et al. evaluated the efficacy of an anti-BCMA CAR-T cell therapy in 20 RR patients (7 of them with EMD involvement). Even if the ORR was not statistically significant between the EMD and not EMD groups (71.43% vs. 80%), patients with EMD achieved a shorter 1-year PFS and OS and developed more AEs in terms of CRS and ICANS [104]. Lastly, in a phase 1b/2 study on Cilta-cel, patients with EMD (N = 13) obtained an ORR comparable to that of the overall study population (94.7%) [105]. 

#### 3.4.3. Recent Updates: What Is Boiling in the Pot?

Updated results on mezigdomide have been recently presented at ASH 2022. Mezigdomize (MEZI) is an oral CRBN E3 ligase modulator that degrades transcription factors (Ikaros and Aiolos) involved in MM pathobiology. The **CC-92480-MM-001 phase 1/2 trial** evaluates MEZI alone or in combination with dexamethasone in RR patients [≥3 prior LOTs (including anti-BCMA therapy) or TCR]. A total of 101 patients received the MEZI+DEX combination. Those affected by EMD (N = 39) achieved a promising ORR of 30.8% [106]. 

Other encouraging data from ASH 2022 come from the study **MonumenTAL-1**: EMD patients treated with the anti-GPRC5D bispecific antibody talquetamab can reach a 50% ORR [107]. 

Even more recently, the **phase Ib RedirectTT-1** trial evaluated the combination anti-BCMA + anti-GPRC5D antibody (teclistamab + talquetamab) in 93 RR patients (66% penta-refractory, 38% with EMD and 33% with HRCAs). The median time to respond was 2 months. In patients with EMD, 85.7% responded to the recommended dose, with 28.6% achieving CR. No new or additive toxicities were reported for both drugs [108]. 

Focusing on recent data from real-life experience, our single-center study presented at the EMN 2023 evaluated 52 patients with EMD (52% at diagnosis and 48% at relapse). Patients were divided into two groups: diagnosed and treated from 1994 to 2012 (group A, N = 11), and from 2013 (group B, N = 41). All were exposed to first line of therapy, 81% to second, 73% to 3rd, 46% to 4th, 33% to >5th, 9% to >8th, 6% received allo-SCT; <50% are still alive but 95% of them belong to the group B (treated after 2012). Moreover, cumulative mOS was 65 months (1–202 months). Despite the generally poor prognosis, these data prove that in the last two decades, there has been an evolution in EMD outcomes [109].

#### 3.4.4. Current Approaches

At the current state, there is no international recommendation for the treatment of EMD and therapeutic choices depend on transplant eligibility or not. Bortezomib-based regimens without or with daratumumab (e.g.: RVd or D-RVd) are a suitable treatment for TE patient with primary EMD. More intensive regimens [RVd/KRd plus cisplatin/doxorubicin/cyclophosphamide/etoposide (PACE)] should be considered in TE patients with proliferative EMD. On the other hand, NTE patients could benefit from other regimens such as D-MPV or D-VCD. lymphoma-like polychemotherapy [PACE/dexamethasone-cyclophosphamide-etoposide-cisplatin (DCEP)/dexamethasone-carmustine-etoposide-doxorubicin-melphalan (Dexa-BEAM)] are mainly employed in the relapse [110]. Lastly, the role of the melflufen-dexamethasone regimen has been evaluated in heavily pre-treated EMD patients in the phase 2 study HORIZON reporting a 22% ORR [111].

Among therapeutic strategies in EMD, a separate case is represented by CNS localization. It represents a rare (<1%) and particularly aggressive (mOS 3.4 months) EMD subcategory especially because of the poor permeability of the blood-brain barrier (BBB) to novel drugs. IMiDs (such as pomalidomide or thalidomide) and PIs such as marizomib [112] have shown a major capability to penetrate this sanctuary site. Combining radiotherapy and/or intrathecal chemotherapy with systemic therapy could be a winning strategy to prolong survival [86].

## 4. Conclusions

Results from RCTs have led to the approval of novel pharmacological combinations which allowed to re-design of the international guidelines. Furthermore, trials have highlighted the primary importance of MRD in guiding treatment decisions. Therefore, it is essential to adopt standardized methodologies to assess MRD at specific time points. No international therapeutic consensus exists for RR disease. However, we can select more appropriate regimens in the first relapse according to prior exposure to lenalidomide or not. Treating further relapses is even more challenging since the use of quadruplets/triplets in early lines has increased the % of TCR patients. Selinexor and belantamab may represent a possible strategy, despite causing a certain toxicity. 

Finally, T-cell immunotherapy (CAR-T, BsAbs) has been proposed and achieved a more durable and profound response in heavily pre-treated patients. Unfortunately, post-CAR-T relapse is frequent. Further biological insights are required to understand treatment failure and guide subsequent strategies. Adopting a sequencing-based approach by using other T-cell therapies has led to surprising results in various phase I and II studies. The role of auto/allo-SCT post-CR-T failure is yet to be defined. Lastly, upcoming agents such as modakafusp alfa and mezigdomide are showing promising activity in the RR setting. 

The extramedullary disease remains a major unmet clinical need and the struggle to find a treatment consensus is still underway. 

Arrived at this point, we are strongly encouraged to test novel agents in real life in order to confirm data obtained from studies and understand the best way to treat those patients who do not meet the eligibility criteria for clinical trials.

## Figures and Tables

**Table 1:** Frailty scores.

SCORE	Geriatric	Biologic	Cytogenetics	Score Range	Interpretation
(Reference)	Domains	Marker	Included?		
**IMWG**	Age	None	**No**	0–2	0 (fit)
[27]	CCI				1 (intermediate fit)
	ADLs				2 (frail)
	IADLs				
**R-MCI**	Age	None	**Yes**	0–9	0–3 (fit)
[28]	Fried Frailty				4–6 (intermediate fit)
	Lung fuction				7–9 (frail)
	Renal function				
	KPS				
**SFS**	Age	None	**No**	0–2	0–1 (not frail)
[29]	CCI				≥2 (frail)
	ECOG-PS				

The table shows 3 major frailty scores: (1) International Myeloma Working Groupo-Frailty Index (IMWG-FI); (2) Revised Myeloma Comorbidity Index (R-MCI); Simplified Frailty Scale (SFS). **ABBREVIATIONS:** CCI, Charlson Comorbidity Index; ADLs, Katz Activity of Daily Living; IADLs, Lawton Instrumental Activity of Daily Living; KPS, Karnofsky Performance Status; HRCAs; ECOG-PS, Eastern Cooperative Oncology Group-Performance Status.

**Table 2:** Staging Systems [29].

Staging	Variables	Cytogenetics	Stage	Criteria	
System		Included?			
**ISS**	Sβ2M	No	I–III	I (Sβ2M < 3.5 mg/L; s.albumin ≥ 3.5 gr/dL)	
	Serum albumin			II (not ISS stage I or III)	
				III (Sβ2M ≥ 5.5 mg/L)	
**R-ISS**	Sβ2M	Yes *	I–III	I (ISS stage I: SR CA by FISH; normal LDH level)	
	Serum albumin			II (not ISS stage I or III)	
	LDH			III (ISS stage III and either HR CA or higher LDH level)	
**R2-ISS**	Sβ2M	Yes **	I–IV	**R2-ISS assigns a score to the following variables:**	
	Serum albumin			ISS II [1], ISS III [1.5], del(17p) [1], high LDH [1],	
	LDH			t (4;14) [1], and amp 1q21 [0.5].	
				**The sum of these scores determines the risk group:**	
				I low (0), II low/intermediate (0.5–1)	
				III intermediate/high (1.5–2.5), IV high (>2.5)	

* del (17p), t (4;14), t (14;16); ** R2-ISS replaces t (14;16) with the amplification 1q21. The table summarizes the principles of staging systems: the International Staging System (ISS); the Revised ISS (R-ISS); ISS secondly revised (2R-ISS). ABBREVIATIONS: Sβ2M, serum-β_2_-microglobulin; LDH, Lactate Dehydrogenase; CA, Cytogenetic Abnormalities; FISH, Fluorescence in Situ Hybridization; HR, High Risk; SR, standard Risk.

**Table 3:** Real Life studies in first relapse.

Authors (Reference)	N° of R/R	Prognostic Factors	Treatment	ORR (and CR + VGPR)	mPFS	mOS
1. Conticello et al., 2019 * [52]	130	ISS III 32%	K-Rd	60% (37%)	HR 8.4 months	78% (2y)
		HRCAs 14.6%			LR NR	with VGPR/CR
2. Palmieri et al, 2020 [53]	123	ISS III 29%	K-Rd	85% (65%)	23 months	33 months
		Cytogenetic NA				
3. Mele et al., 2021 ** [54]	130	ISS 42%	K-Rd	79% (91%)	54% (2y)	70% (2y)
		HRCAs 6.2%				
4. Antonioli et al, 2020 [55]	44	ISS III 24%	D-Rd	79% (55%)	HR 19% (1y)	81% (1y)
		HRCAs 34%			SR 90% (1y)	
5. Fazio et al., 2022 [56]	171	ISS III 24%	D-Rd	93% (81%)	77% (1y)	84% (1y)
		Cytogenetic NA	vs			
			D-Vd	76% (49%)		

* Patients who received the full-dosage of carfilzomib in the first 2 cycles had a better outcome. ** 21 patients performed ASCT after K-RD achieving a 2y-PFS of 100%. Thus, K-Rd should be considered as a bridge to ASCT. The table outlines the results of some recent Italian real-life studies: (1) A retrospective real-life survey of the Sicilian Myeloma Network; (2) A retrospective study at 14 different institutions of Southern Italy; (3) The real-life experience of Rete Ematologica Pugliese; (4) Single-center experience, Haematology Unit, Careggi Hospital of Florence; (5) Real-life survey of Sicilian Myeloma Network. ABBREVIATIONS: R/R, relapsed refractory; ISS, International Scoring System; HR, High Risk; HRCAs, High-Risk Cytogenetic Abnormalities; SR, Standard Risk; K, carfilzomib; R, lenalidomide; D, daratumumab; V, bortezomib; d, dexamethasone; ORR, Overall Response Rate; CR, Complete Response; VGPR, Very Good Partial Response; NR, Not Reached; NA, Not Assessed; mPFS, median Progression Free Survival; OS, median Overall Survival; NR, Not reached; y, year; m, months.

**Table 4:** Real-life studies in heavily pre-treated patients.

Authors	R/R	Prognostic	n° Prior	Treatment	ORR	mPFS (Months)	mOS (Months)
	Patients	Factors	LOTs				
1. Mateos et al., 2022 [69]	248	PS 0–1	4 (2–13)	92 varied	29.8%	4.6 *	12.4
		IS III 35%		SOC regimens			
2. Morabito et al, 2022 [70]	883	ISS III	1 (1–11)	Elo-Rd	Elo-Rd 53.9%	20.3	33.4
		29.1% **		vs. K-Rd	K-Rd 37%		
3. Markovic et al, 2021 [71]	44	HRCA 60%	4 (2–9)	DARA	VGPR > 27%; PR 10%	7.2 ***	7.8
				single agent	MR 14%; SD 22%		
4. Hose et al., 2021 [72]	22	ISS III 22.7%	5 (1–16)	Elo-Pd	50%	13.5 (ISS I–II)	NR
		HRCA 18%				6.4 (ISS III)	(42.5 m FUP)

* The 92 varied regimens utilized demonstrate a lack of clear SOC for heavily pretreated, triple-class exposed patients with RRMM in real-world practice. As a result, outcomes are generally poor. ** ISS III, LENA exposure, age > 65 y, and n° of prior lines were predictors of worse prognosis. *** In univariate analysis, PFS and OS resulted in superior if patients obtained PR within 6 months. Furthermore, PFS was longer in those previously treated with PD rather than K-Rd (9.3 vs. 3.4 months). The table shows an overview of some national and European real-life studies including heavily pre-treated patients: (1) LocoMMotion is the first prospective study of real-life SOC in triple-class exposed patients (at least a PI, IMiD, and anti-CD38 mAb); (2) An Italian multicenter experience; (3) A retrospective real-life survey of the Sicilia Myeloma Network; (4) A real-life experience from the German-Austrian group. ABBREVIATIONS: LOTs, Line OF Therapy; ORR, Overall Response Rate; mPFS, median Progression Free Survival; mOS, median Overall Survival; SOC, Standard of Care; ISS, International Scoring System; HRCAs, High-Risk Cytogenetic Abnormalities; CR, Complete Response; VGPR, Very Good Partial Response; PR, Partial Response; MR, Major Response; SD, Stable disease; NR, Not Reached.
